# Primary Testicular Cutaneous Metastasis: A Clinical and Dermoscopic Diagnostic Challenge

**DOI:** 10.7759/cureus.105125

**Published:** 2026-03-12

**Authors:** Karen Uriarte-Ruiz, Emilio Peniche-Luna, Amairani Manriquez-Robles, Elisa Vega-Memije, Cristina Berumen-Glinz

**Affiliations:** 1 Department of Dermatology, Hospital General "Dr. Manuel Gea González", Mexico City, MEX

**Keywords:** cutaneous dermal metastasis, neoformations, non-seminomatous germ cell tumors, oncodermatology, testicular carcinoma

## Abstract

This article aimed to report a sporadic case of a primary testicular cutaneous metastasis, which represents a clinical and dermoscopic diagnostic challenge due to its low prevalence and non-specific dermoscopic presentation. A 24-year-old male with a history of primary non-seminomatous germ cell tumor of the left testis presented with a disseminated dermatosis characterized by raised, dome-like lesions. On dermoscopy, the lesions presented with milky-red areas, blue-white veil-like zones, multiple blood crusts, and structureless white areas. The heterogeneous morphology of the lesion raised suspicion for malignancy. A skin biopsy was performed, confirming the diagnosis of cutaneous intralymphatic infiltration by a poorly differentiated carcinoma. Cutaneous metastasis from a primary testicular tumor is exceptionally rare. Although recent efforts have improved the dermoscopic characterization of cutaneous metastases, standardized diagnostic criteria have not yet been fully established. Cutaneous metastases indicate advanced disease and carry significant prognostic implications. Such dermoscopic findings may contribute to the recognition of cutaneous signs that prompt further oncologic evaluation or suggest disease progression, particularly when clinical manifestations are misleading.

## Introduction

Cutaneous metastases from non-seminomatous testicular tumors are exceptionally rare, with an estimated incidence of 0.04% among urologic malignancies and fewer than 12 cases reported since 1989 [1]. This low prevalence renders it an extraordinary finding that warrants specialized and multidisciplinary attention.

They commonly present as nodular lesions involving the head, neck, and trunk, and most frequently spread via hematogenous dissemination. The raised, dome-shaped, pink-violaceous morphology, clinically simulating Kaposi sarcoma or benign lesions as hemangiomas, poses a significant diagnostic challenge for dermatologists. This case underscores the importance of thorough clinical examination, dermoscopy, and timely biopsy in order to accurately distinguish between benign vascular neoplasms and malignant metastatic lesions.

## Case presentation

A 24-year-old male came to our inpatient consultation with a history of primary non-seminomatous germ cell tumor of the left testis, clinical stage IIIC, with pulmonary metastatic disease, currently receiving third-line chemotherapy with gemcitabine and oxaliplatin, who underwent retroperitoneal lymphadenectomy in July 2024.

**Figure 1:**
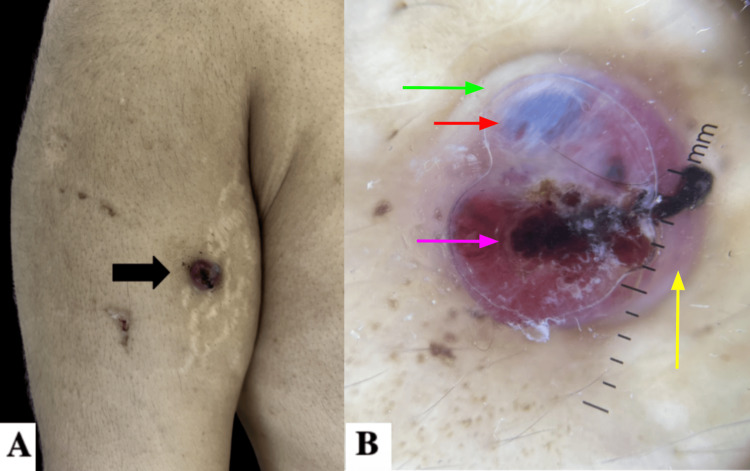
Nodular lesion on the right arm (A) Nodular violaceous lesion with well-defined, regular borders on the right arm (arrow), along with a superficial hemorrhagic crust. (B) On dermoscopy, the same lesion, approximately 10 mm in diameter, is characterized by milky-red areas (yellow arrow), blue-white veil–like zones (red arrow), multiple blood crusts (pink arrow), and structureless white areas (green arrow).

**Figure 2:**
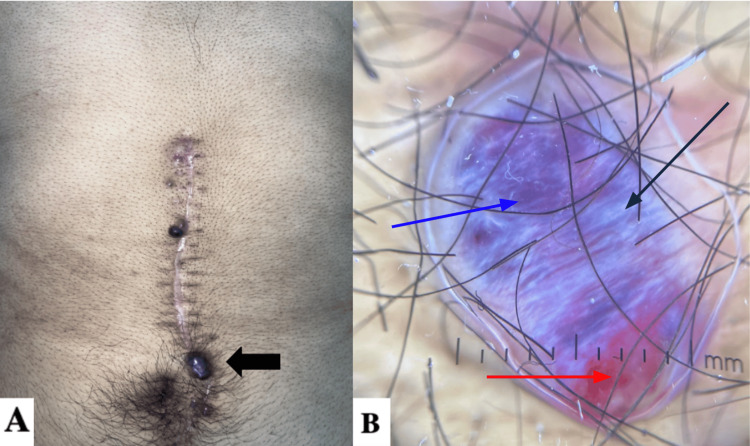
Nodular lesion on the midline abdomen A) A well-circumscribed pink-violaceous nodule, dome-shaped, with a smooth, shiny surface is observed along a midline abdominal surgical scar. B) On dermoscopy, the same lesion, approximately 10 mm in diameter, arises from a structureless base, displaying a homogeneous pink area (red arrow). However, blue-white veil (blue arrow) and shiny white lines (black arrow) are absent in angiomas and other benign lesions.

One month following the lymphadenectomy, he developed a disseminated dermatosis affecting the face, nasal tip, right arm (Figure 1), and midline abdomen (Figure 2). The lesions were characterized by raised, dome-shaped neoplasms with regular, well-defined borders and a pink-violaceous coloration, varying in size from 0.5 to 1 cm in diameter. On dermoscopy, the lesions presented with milky-red areas, blue-white veil-like zones, multiple blood crusts, and structureless white areas. The heterogeneous morphology of the lesion raised suspicion for malignancy.

**Figure 3:**
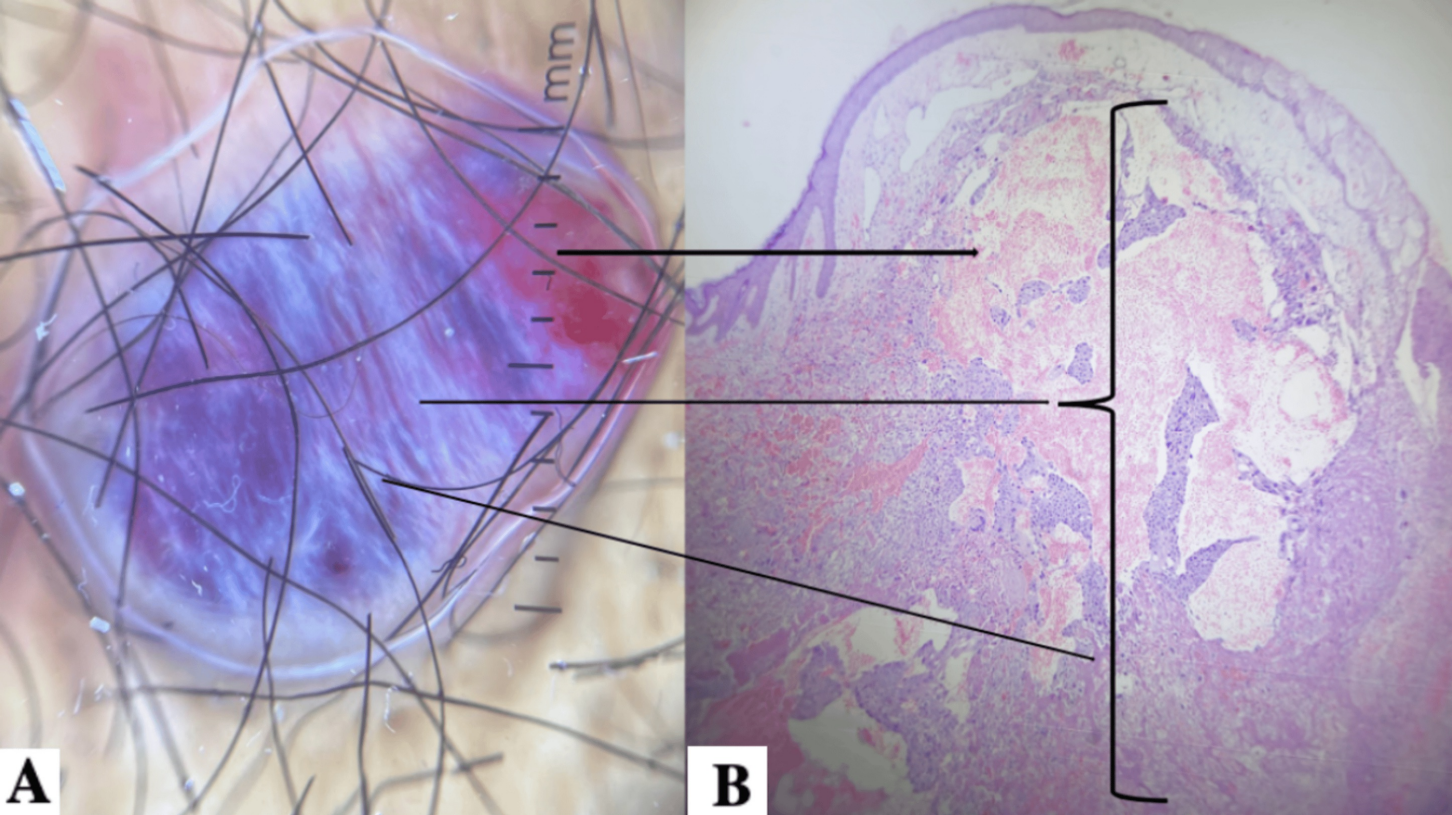
Comparison of ectatic vessels in dermoscopy and histologic findings A) Dermoscopic view of the abdominal lesion. The upper arrow shows a red homogeneous background compatible with dilated superficial vessels, while the mid and lower arrows show a deeper violaceous area representing underlying vascular congestion. B) Histologic section of the abdominal lesion. The upper arrow connects with the presence of ectatic vessels and extensive hemorrhagic areas in the dermis. The mid and lower arrow correspond to tumoral infiltration and vascular dilation which give the bluish color.

**Figure 4:**
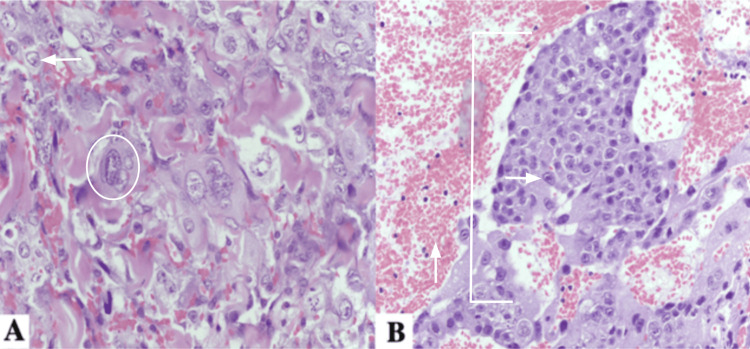
Histologic findings compatible with a diagnosis of poorly differentiated carcinoma A) Malignant tumor cells with abundant eosinophilic cytoplasm, enlarged hyperchromatic nuclei, and prominent nuclear atypia (arrow). Frequent atypical mitotic figures are present (circle), consistent with a high-grade malignant neoplasm B) Presence of solid nests (bracket) and malignant epithelial cells with nuclear pleomorphism and prominent nucleoli (horizontal arrow), embedded within a hemorrhagic dermal stroma (vertical arrow).

A skin biopsy of a lesion on the right arm was performed with differential diagnoses including Kaposi sarcoma, hemangioma, and cutaneous metastasis. Histopathological examination revealed a germ cell-derived neoplasm composed of large cells with abundant, dense, eosinophilic cytoplasm, pleomorphic nuclei, and numerous atypical mitotic figures from the papillary dermis to the subcutaneous tissue. Dissected collagen bundles, vascular wall invasion, and alternating areas of necrosis were observed. In addition, multiple ectatic vessels lined by a thin layer of endothelial cells and extensive areas of hemorrhage were identified (Figure 3). Dissected collagen bundles, vascular wall invasion, and alternating areas of necrosis were observed. In addition, multiple ectatic vessels lined by a thin layer of endothelial cells and extensive areas of hemorrhage were identified (Figure 4). A diagnosis of intralymphatic infiltration by poorly differentiated carcinoma involving the superficial and mid dermis was established. Following histopathological confirmation, the patient’s clinical condition rapidly deteriorated. Laboratory evaluation revealed severe anemia, with a hemoglobin level of 4 g/dL. Owing to his critical clinical status and the inability to perform transfusion support in a timely manner, no additional oncologic intervention could be initiated. The patient died within 24 hours of diagnosis. 

## Discussion

This case represents a significant diagnostic challenge due to its clinical morphology and dermoscopic features, which closely resemble other entities such as Kaposi sarcoma and vascular tumors.

Cutaneous metastases most frequently arise from breast, colorectal, and lung carcinomas, as well as melanoma, and are generally associated with a poor prognosis. The reported incidence of cutaneous metastasis in urologic malignancies ranges from 0.73% to 1.3% [2,3]. Among these, renal cell carcinoma is the most common subtype, followed by bladder, prostate, and testicular carcinoma, the latter accounting for approximately 0.04% [4]. Cutaneous metastasis from testicular tumors is exceedingly rare and has been reported primarily in non-seminomatous germ cell tumors, particularly choriocarcinoma, while seminomas and sex-cord stromal tumors only rarely involve the skin [1].

Although recent efforts have improved the dermoscopic characterization of cutaneous metastases, standardized diagnostic criteria have not yet been fully established. However, six dermoscopic patterns have recently been described to assist in clinical orientation: vascular, heterogeneous, structureless, blue nevus-like, nevus-like, and angioma-like patterns [5-7]. In the present case, the dermoscopic findings were consistent with an angioma-like pattern, characterized by dilated blood vessels and pink homogeneous areas with blotches. However, the presence of structureless base, shiny white lines, and blue-veil areas was suggestive of cutaneous metastatic lesions.

Cutaneous metastases indicate advanced disease and carry significant prognostic implications. In this patient, cutaneous dissemination developed after multiple lines of chemotherapy and retroperitoneal lymphadenectomy, reflecting systemic disease progression.

The presence of intralymphatic dermal infiltration by poorly differentiated germ cell tumor cells, characterized by atypical mitoses, necrosis, and extensive hemorrhage, represents an unusual and educational finding. These features provide a valuable opportunity for clinicopathologic correlation in oncologic dermatology. Notably, up to 22% of cutaneous metastases may precede the diagnosis of the primary tumor, underscoring the crucial role of the dermatologist as an early detector of internal malignancies, particularly when clinical manifestations are misleading.

## Conclusions

Although uncommon, cutaneous metastases should be considered in the differential diagnosis of exophytic lesions in patients with a history of prior malignancy. Dermoscopy serves as a useful adjunctive tool to guide clinical suspicion; however, definitive diagnosis requires histopathologic confirmation through biopsy. Cutaneous metastases represent a clinical and dermoscopic challenge, where features may aid in identifying cutaneous signs that prompt further oncologic evaluation or indicate disease progression, particularly in atypical presentations.

## References

[1] Elkeeb D, Hopkins Z, Florell SR (2018). Pure testicular choriocarcinoma presenting as a friable hemorrhagic nodule on the lip. JAAD Case Rep.

[2] Toberer F, Enk A, Hartschuh W, Grüllich C (2018). Testicular choriocarcinoma with cutaneous metastasis in a 19-year-old man. J Cutan Pathol.

[3] Menon AR, Thomas AS, Suresh N, Shashidhar SM (2016). Cutaneous metastasis: an unusual presenting feature of urologic malignancies. Urol Ann.

[4] Mueller TJ, Wu H, Greenberg RE, Hudes G, Topham N, Lessin SR, Uzzo RG (2004). Cutaneous metastases from genitourinary malignancies. Urology.

[5] Chernoff KA, Marghoob AA, Lacouture ME, Deng L, Busam KJ, Myskowski PL (2014). Dermoscopic findings in cutaneous metastases. JAMA Dermatol.

[6] Deinlein T, Haenssle HA, Fink C, Hofmann-Wellenhof R, Blum A (2019). [Dermoscopy of cutaneous metastases]. Hautarzt.

[7] Tiodorovic D, Stojkovic-Filipovic J, Marghoob A (2024). Dermatoscopic patterns of cutaneous metastases: a multicentre cross-sectional study of the International Dermoscopy Society. J Eur Acad Dermatol Venereol.

